# The Spatial Limitations of Current Neutral Models of Biodiversity

**DOI:** 10.1371/journal.pone.0014717

**Published:** 2011-03-14

**Authors:** Rampal S. Etienne, James Rosindell

**Affiliations:** 1 Community and Conservation Ecology Group, Centre for Ecological and Evolutionary Studies, University of Groningen, Groningen, The Netherlands; 2 Institute of Integrative and Comparative Biology, University of Leeds, Leeds, United Kingdom; University of Oxford, United Kingdom

## Abstract

The unified neutral theory of biodiversity and biogeography is increasingly accepted as an informative null model of community composition and dynamics. It has successfully produced macro-ecological patterns such as species-area relationships and species abundance distributions. However, the models employed make many unrealistic auxiliary assumptions. For example, the popular spatially implicit version assumes a local plot exchanging migrants with a large panmictic regional source pool. This simple structure allows rigorous testing of its fit to data. In contrast, spatially explicit models assume that offspring disperse only limited distances from their parents, but one cannot as yet test the significance of their fit to data. Here we compare the spatially explicit and the spatially implicit model, fitting the most-used implicit model (with two levels, local and regional) to data simulated by the most-used spatially explicit model (where offspring are distributed about their parent on a grid according to either a radially symmetric Gaussian or a ‘fat-tailed’ distribution). Based on these fits, we express spatially implicit parameters in terms of spatially explicit parameters. This suggests how we may obtain estimates of spatially explicit parameters from spatially implicit ones. The relationship between these parameters, however, makes no intuitive sense. Furthermore, the spatially implicit model usually fits observed species-abundance distributions better than those calculated from the spatially explicit model's simulated data. Current spatially explicit neutral models therefore have limited descriptive power. However, our results suggest that a fatter tail of the dispersal kernel seems to improve the fit, suggesting that dispersal kernels with even fatter tails should be studied in future. We conclude that more advanced spatially explicit models and tools to analyze them need to be developed.

## Introduction

The neutral theory of biodiversity has received much attention over the last decade since Hubbell published a comprehensive summary of it in 2001 [1]. While initial reviewers criticized its assumption of functional equivalence of all individuals (regardless of species) in an ecological community [2]–[5], it is now increasingly acknowledged that it serves well as a null model of community composition and dynamics [6]–>[9], playing a role similar to the neutral model of molecular evolution [10] in population genetics, both models being good examples of “significant” theories based on unrealistic assumptions [11]. Furthermore, some mismatches between the theory's predictions and empirical data may have no connection with neutrality, but may instead reflect auxiliary assumptions. In the simple, most widely used, mainland-island model [1], [12] two such assumptions stand out. First, it models space implicitly by assuming a local community receiving immigrants from a larger panmictic source pool (metacommunity [1]) where speciation balances extinction. Second, for mathematical convenience it models speciation as simple point mutation. To overcome the limitations of these auxiliary assumptions, the model has been extended to include different speciation modes, such as random fission [13], [14], abundance-independent speciation rates [15], [16] and protracted speciation [17]. Also, models with different spatial structures have been investigated, such as extensions of the simple model with multiple local communities (rather than a single community) connected to the same metacommunity [18]–[22], patch models [23], [24] and even completely spatially explicit models [25]–[30]. Although the spatially explicit models are the most natural and consistent way to model birth, death, speciation and dispersal processes, their difficulty allows limited analytical treatment only (for example only for specific dispersal kernels [30] and so far only for species turnover and species-area relationships, but not for species abundance distributions), seriously diminishing the ease of statistical comparisons to data. An attractive feature of the spatially implicit model (with one or more local communities) is its analytical tractability and its amenability to data comparison through the use of sampling formulas [31], [32]. Yet it contains an inconsistency [33] the implications of which have rarely been examined: while local communities can be dispersal limited, the metacommunity - which is the collection of all local communities [1] - is assumed to be panmictic, i.e. homogeneously mixed, without dispersal limitation. This does not necessarily mean that all results obtained with this model are false, but the inconsistency is certainly a reason for concern. In this paper, we ask whether, and if so how, a spatially explicit model can be approximated by a spatially implicit model. Particularly, we want to interpret the rather abstract parameters of the spatially implicit model (the fundamental biodiversity number 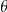, a measure of the diversity of the panmictic source pool, and fundamental immigration number 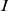, a measure of the potential of an individual from this source pool to immigrate into a local community), in terms of the biologically more sensible parameters of the spatially explicit model (speciation rate 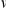 and dispersal distance 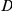, a measure of the mean and median dispersal distance, see also Figure S1). We do this by fitting the most widely used spatially implicit model [1] to abundance distributions generated by simulations, using coalescence, of a spatially explicit model with a radially symmetric dispersal kernel; we explore both the mathematically convenient Gaussian dispersal kernel [27], [30] and a more realistic fat-tailed dispersal kernel [28].

## Results

The regression of 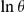 and 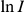 versus 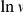 and 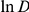 is extremely good with 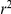-values greater than 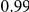 for all simulation sets. This means that 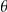 and 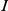 can be approximated by power laws of 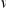 and 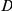 (for the parameter ranges that we simulated):
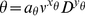
(1a)

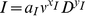
(1b)which means that the surfaces in 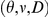-space and 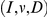-space are almost planar in logarithmic space (Figure 2). The exact regression coefficients are shown in Table 1. Eqns (1a)–(1b) imply that 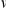 and 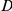 can, in principle, be expressed as functions of 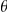 and 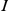:
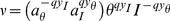
(2a)

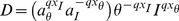
(2b)where
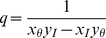
(3)


**Table 1:** Coefficients and 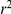-values of the regression of 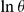 and 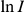 versus 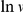 and 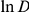 for various sizes and shapes of samples from the spatially explicit model results.

Simulation type	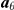	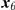	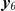	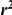	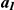	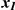	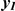	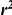
Gaussian dispersal kernel								
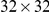								
square sample	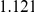	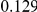	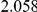	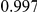	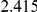	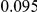	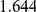	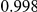
circular sample	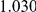	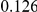	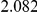	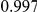	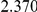	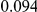	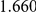	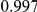
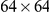								
square sample	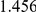	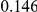	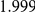	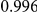	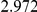	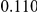	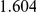	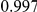
circular sample	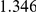	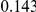	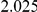	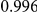	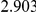	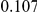	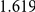	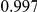
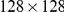								
square sample	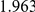	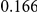	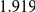	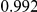	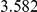	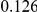	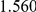	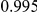
circular sample	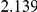	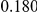	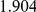	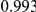	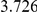	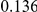	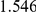	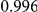
Fat-tailed dispersal kernel								
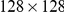								
circular sample	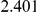	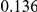	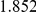	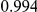	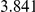	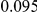	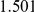	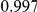

While our results suggest that there is a clear correspondence of the *parameters* of the spatially explicit and spatially implicit models, the models themselves do not show an exact match as evidenced by the goodness-of-fit (GOF) results shown in Figure 3 for each parameter combination of the spatially explicit model. For the more reasonable values of the speciation rate 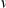, the GOF is around 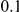, except for a small range of values of the dispersal distance 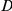 where it is higher.

The regression coefficients suggest that 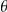 and 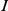 may be highly correlated. Indeed, Figure 4 shows that many of the estimated parameter combinations fall on or around a single straight line. The points that fall on the line are usually the ones with lower GOF, whereas points further from the line have higher GOF. Remarkably, the parameter estimates for the six tropical forest plots studied by [34] and others, all fall well above the line. Studying the figure more closely, the points in 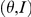-space that give the same number of species lie on concave curves from top left to bottom right. This suggests that points on the line merely fit the species richness, whereas points off the line are more likely to fit the full abundance distribution.

We explored the GOF further by looking at the abundance distributions for several points both on and off the line; we selected two points for each 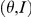-parameter combination: one with low GOF and one with high GOF, indicated in Figure 4 with red circles. The fit seems fair regardless of the GOF-value (Figure 5) but one observes that for low GOF-values the rank abundance curve (Figure 6) is among the lowest for some ranks and among the highest for others, resulting in an overall poorer fit.

These results are all for the Gaussian dispersal kernel. We repeated the regression analysis for spatially explicit simulations in the 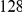 by 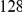 circular sample with a fat-tailed kernel (see Methods). We found somewhat lower values of the exponents 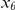, 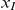, 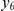 and 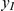 and somewhat higher prefactors 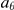 and 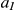 than in the Gaussian case, but qualitatively the results are similar (Table 1, Figures S2, S3, S4). The GOF seems to be slightly higher than in the Gaussian case, and there seem to be more points off the diagonal of the 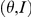-graph which have a relatively high GOF.

## Discussion

We have shown that the parameters of the spatially explicit and the spatially implicit neutral models are related to one another by a simple power law. This means that estimates of the compound parameters 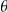 and 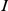 of the spatially implicit model, obtained by maximization of the likelihood given by the analytical sampling formula, can be converted to the ecologically more sensible parameters of 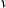 and 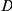. The conversion seems to be better for smaller and circular samples. However, the correspondence makes no intuitive sense: both 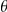 and 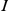 depend only slightly on the speciation rate and much more on the dispersal distance.

While the parameters of the spatially explicit and implicit models are clearly related by a power law and the fit between the spatially implicit and explicit models is a reasonable approximation to the eye, a rigorous statistical comparison tells us that there are differences between the two. For the more realistic values of the speciation rate 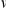, the goodness-of-fit (GOF) falls below 10% where 50% would be obtained if the data were actually generated by the spatially implicit model itself. Because the spatially explicit model is not identical to the spatially implicit model, it can be expected that the GOF will not be high, but even in this light 10% is not an amazing performance. The best GOF-values are observed for 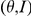-combinations that are off the diagonal line formed by most of the parameter combinations. This is also where the estimates for real data are found.

The fact that most 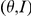-combinations fall on a line means that not all parameter combinations of the spatially implicit model are easily accessible for the spatially explicit model, and the less accessible parameter combinations seem to be the ones usually obtained for real data as suggested by the fitted parameters of the tropical forest data sets. A possible explanation for this is that the mainland-island structure of the spatially implicit model allows the system to be more isolated than can be captured by the spatially explicit model. It is important to note that the reduced accessibility is due to the difference in structure of the species abundance distribution, not due to different predictions of species richness, because curves of equal species richness can cover the 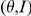-space quite well.

With the likelihood-based GOF-test [18] we observed that fits appearing to be fair from visual inspection of the abundance distribution can have low goodness-of-fit values. Hence, visual GOF tests using binned abundance distributions are largely uninformative unless the visual fit is bad, in which case the statistical fit will be bad too. Rank-abundance distributions contain the full abundance vector of a sample and are therefore more informative. This has been argued before (e.g. [35]), but nevertheless binned SADs are still widely used. Perhaps our results will help to make this point again. Nevertheless, our results show that one needs to be extremely careful in assessing GOF from visual inspection of the rank-abundance distribution as well. At the same time, one would not expect two different models to be indistinguishable. We may interpret the lower but not extremely low GOF-values as indicating that the spatially explicit and implicit models are behaving differently, but still are a fair match.

Etienne [12] and more rigorously Chisholm & Lichstein [8], provided formulas to compute the immigration parameter 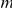 analytically in an independent way using the dispersal kernel. We have shown here with the spatially explicit model - which also uses this dispersal kernel - that fitting the spatially implicit model does not at all provide 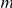-values that are in line with this interpretation. The parameters of the spatially implicit model do not have the interpretation initially thought. For example, 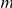 (or 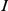) is indicative of dispersal limitation, but also contains information on speciation. Conversely, this means that one cannot so easily refute the validity of the original model based on the original interpretation of its parameters. Therefore, arguments that 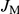 will have to be too large in order to get the observed 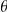 with sensible 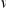 [36] are no longer relevant (see also [17] for an alternative, complementary, argument).

In sum, the spatially explicit and implicit models do have some correspondence, but also behave in substantially different ways, as one would expect. The spatially implicit model often fits species abundance data remarkably well, and our results show that the intuitively more realistic spatially explicit model cannot do so in its current form, as it seems unable to access the parts of parameter space that the parameters of fits of the spatially implicit model to real data occupy. There are two possible explanations for this: either the spatially implicit model only fits reality by pure chance or the most commonly used spatially explicit neutral model is lacking in some way. In the second, more speculative explanation, the spatially implicit model is doing well because it fits something bigger which we cannot see. This may be due to a general property of mean-field models compared to more detailed models. By ignoring most complexity, mean field models actually work better, whereas more detailed models have to get the details right: complexity contingent on other complexity is necessary to make them work, and we have to be extremely careful to get the optimal balance. In our case the spatially explicit model may need to get the results right on the dispersal kernel, by using, for example, dispersal kernels with even fatter tails [28], asymmetric dispersal kernels or kernels that are not centered around the source, or by using finite landscapes. Our results suggest an upward trend in GOF and a downward trend in the exponents 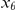 and 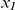 with increasing fatness. We conjecture that this is due to fat-tailed kernels bringing in additional species, thereby allowing a lower speciation rate for the same fit to data as a Gaussian kernel with unrealistically high speciation rate [28]. If the trends persists, much fatter tails might produce a good fit for realistic values of the speciation rate and the dependence of 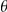 and 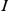 on the speciation rate might disappear altogether. Unfortunately, simulations with fatter-tailed kernels are currently not feasible and analytical treatment remains elusive. All this highlights the importance of spatial processes even in a neutral world and it calls for more research into spatially explicit models, both analytically and with simulutions, particularly with fat-tailed dispersal kernels. The spatially implicit model continues to be the best for fitting to abundance data but in the light of our results the parameters must now be interpreted with great care.

## Methods

### 0.1 Spatially explicit model

We simulated the spatially explicit neutral model in infinite area [27] with simulation procedures based on coalescence as described in [29]. Here we briefly summarize the model's main properties. The spatially explicit model describes processes in an infinite landscape on a grid with each cell being occupied by one individual. Each individual has the same probabability of dying at the next time-step, independently of its fellows: each death is instantly replaced by the immediately maturing off-spring of another individual. Density is therefore kept constant: the zero-sum assumption [1], some relaxations of which leave many of a neutral model's predictions unaltered [37]–[39]. A newly established individual has probability 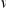 of being an entirely new species. Which individual replaces the deceased individual, depends on the dispersal kernel (i.e. a probability density function.for the location to where the individual disperses). We assumed the following two-dimensional radially symmetric dispersal kernel [40], [28]:
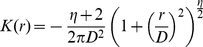
(4)where 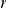 is the distance from the source, 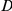 is proportional to the mean and median dispersal distance (it is measured in units of distance between cells, and is equivalent to 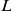 in [28], see Figure S1) and 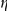 measures the fatness of the tail of the distribution. In most of our simulations we took 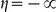 which corresponds to a Gaussian dispersal kernel [40], but because fat-tailed dispersal kernels give more sensible results [28], we repeated the analysis for a fat-tailed kernel with 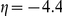. Making the tails even fatter than 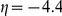 would dramatically increase simulation time because long-distance dispersal postpones coalescence events.

The equilibrium of this model can be elegantly and very efficiently simulated with coalescence methods [29], which follow a local sample from the metacommunity backward rather than forward in time. We ran simulations for samples of various sizes (area 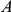 was set at 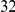 by 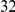, 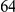 by 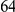 and 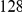 by 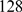) and for a range of parameter values for 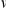 and 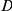: 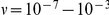 and 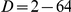 with logarithmic intervals. For each parameter combination, we simulated the equilibrium abundance distribution 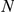 times, where 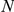 was chosen such that we expect to be able to resolve for the number of species in each abundance class to within an error of 3% with a 99% certainty, with a minimum of 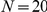 simulations. The required 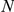 was calculated on the basis of variation in species richness of the first 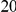 simulations; in practice 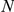 was on average around 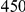.

It has been suggested [8] that circular samples might be more appropriate than squares for this comparison, so we did two sets of simulations: one with squares, the other with circles, both with the same number of individuals (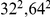 or 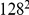). Because for small areas fat-tailed dispersal kernels will give results that are almost identical to those of a Gaussian kernel, only using a smaller speciation rate [28], we only performed simulations with the fat-tailed kernel for the circular area of size 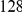 by 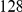.

### 0.2 Spatially implicit model

We used the standard spatially implicit model described by [1] but with overlapping generations, see [32]. The sampling formula for this model was presented by [12]. A panmictic plot of 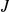 individuals exchanges individuals with a panmictic source pool (the metacommunity). At each time-step, one individual is chosen at random to die Another is chosen at random, with probability 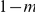 from the plot, and with probability 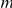 from the metacommunity, to produce the immediately maturing replacement. The metacommunity behaves similarly to the local community, except that it contains 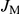 individuals and a death is immediately followed by colonization of offspring of another individual in the metacommunity which can be of a new species due to point mutation (with probability 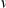) or of an already existing species (with probability 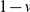). It can be shown [12] that under this model the probability of a particular species abundance distribution of a local community sample of size 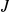 depends on the so-called fundamental biodiversity number 
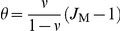
 and the fundamental immigration number 
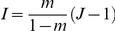
.

### 0.3 Model comparison

**Figure 1:**
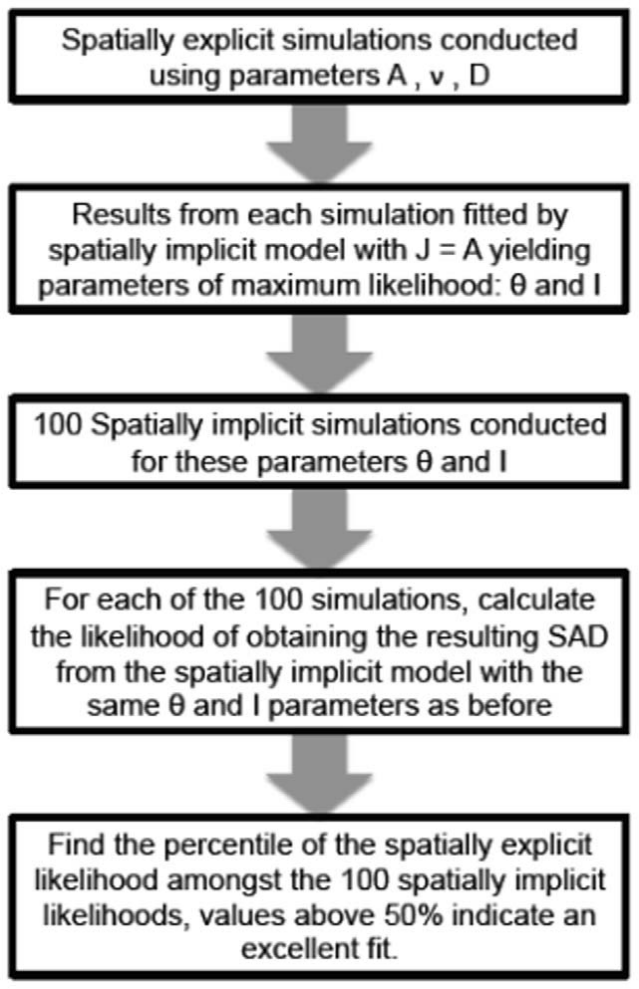
Schematic overview of the methodology to compare the spatially explicit and spatially implicit models.

We compared the spatially explicit and spatially implicit models in the following way. For each species abundance distribution simulated with the spatially explicit model we estimated the parameters 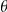 and 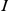 using the sampling formula for the spatially implicit model. Because there may exist two local likelihood optima [41], we started our estimation with two extreme initial conditions and then took the parameter combination 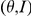 with the highest likelihood. We calculated basic statistics (mean, median, variance) of these parameters for each set of simulation parameters 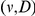. We then regressed the log of the mean or median of 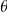 and 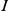 against the logs of 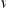 and 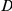 (mean and median gave similar results). To assess goodness-of-fit of the spatially implicit sampling formula to the spatially explicit simulation results, we performed the goodness-of-fit (GOF) test of [18]. To this end we first generated, using the spatially implicit model [12], 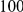 species abundance data sets with each estimated parameter combination 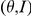. Subsequently we computed the loglikelihoods of the 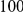 simulated data sets given the estimated parameters [12]. We then compared the loglikelihood of the spatially explicit data with the 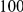 loglikelihoods of the spatially implicit data, and recorded the percentile of the spatially explicit loglikelihood amidst the spatially implicit data. To obtain an overall GOF measure for each parameter combination 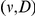 we computed a mean GOF, averaged over the first 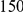 spatially explicit data sets corresponding to the same 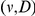-combination. To interpret this GOF measure, note that one would expect a GOF of, on average, 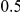 for data that is actually generated with the model for which one is measuring the GOF (we tested this prediction for data generated with the spatially implicit model). Figure 1 shows our procedure schematically.

**Figure 2:**
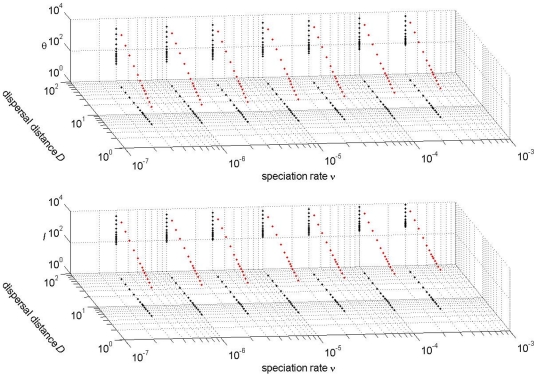
The estimated median values of 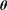 (top panel) and 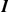 (bottom panel) as a function of speciation rate 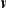 and dispersal distance 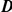. The red dots are the estimated values, the black dots represent the projections onto the null-planes of the 3-dimensional space. The figure is for a circular sample with 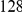 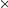 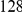 individuals and a Gaussian dispersal kernel, but other samples are very similar. See Figure S2 for analogous results with the fat-tailed dispersal kernel.

**Figure 3:**
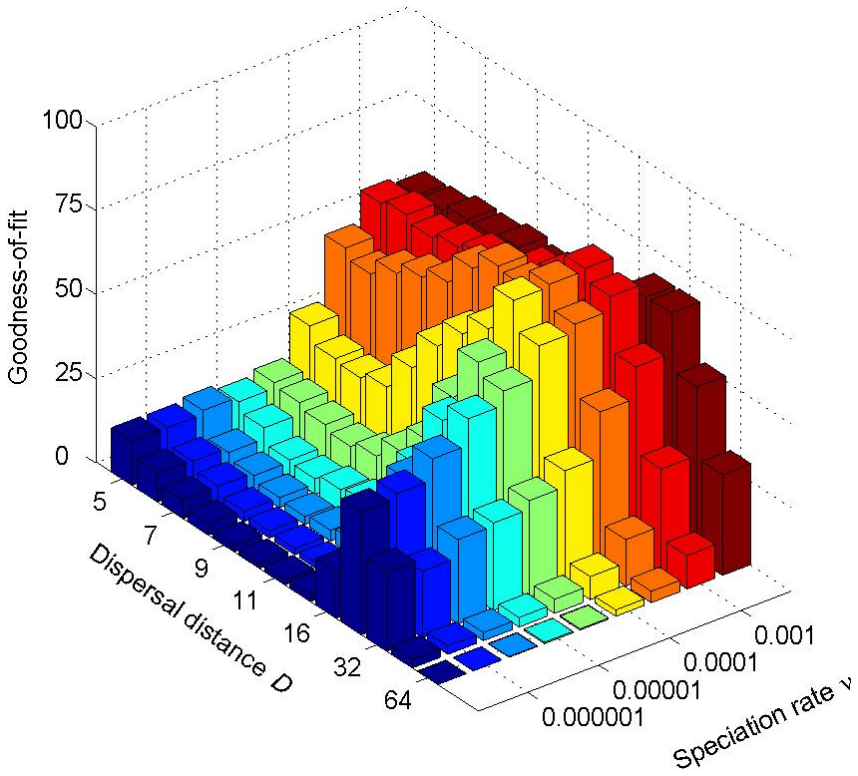
Goodness-of-fit of the spatially implicit model to the species abundance data generated by the spatially explicit model with Gaussian dispersal kernel for various parameter combinations. See Figure S3 for analogous results with the fat-tailed dispersal kernel.

**Figure 4:**
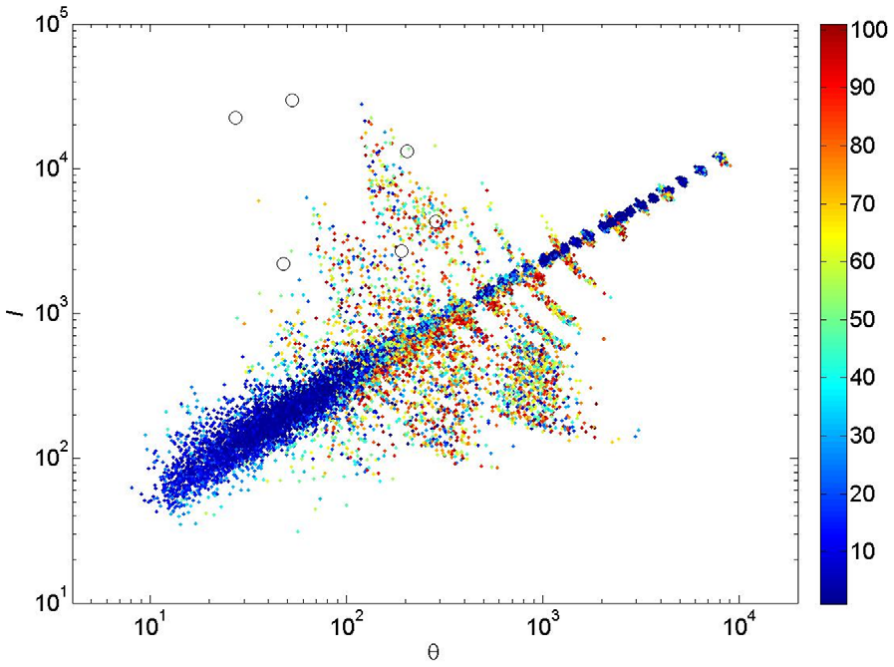
Plot of the estimated 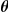-value versus the estimated 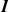-value for all simulations for which a goodness-of-fit was computed. The color of each point indicates the goodness-of-fit, which is expressed as a percentile as colorcoded in the sidebar. The figure is for a circular sample with 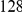 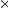 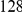 individuals and a Gaussian dispersal kernel, but other samples are very similar. The black circles represent the estimated 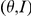-combinations for the six tropical forest plots of [34]. The red circles are needed for Figure 5 and explained in its caption. See Figure S4 for analogous results with the fat-tailed dispersal kernel.

**Figure 5:**
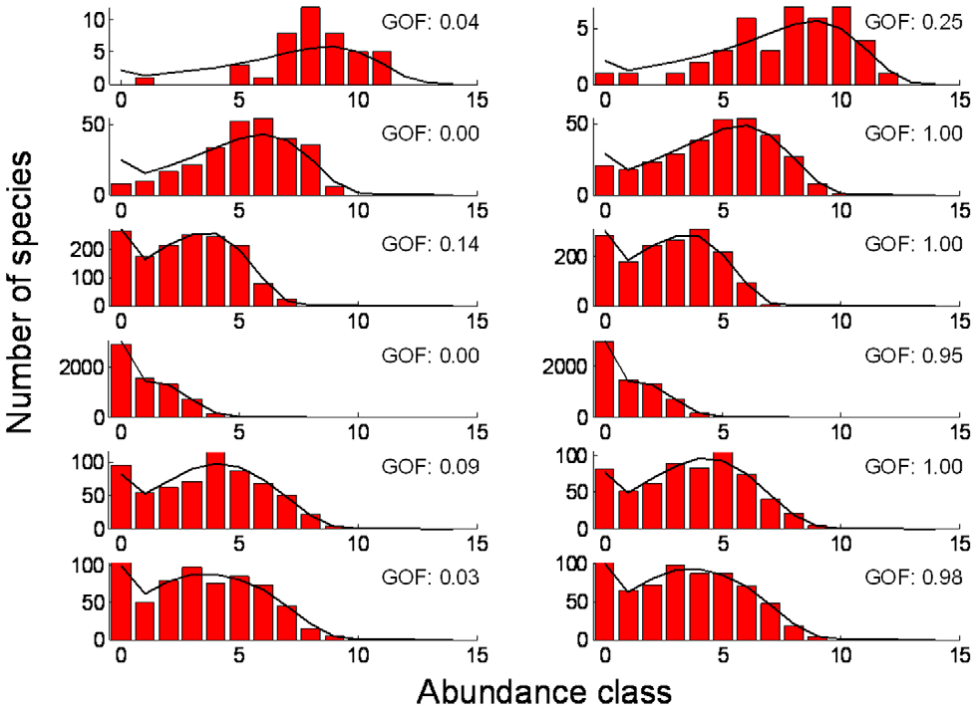
Species abundance distributions for the points indicated with red circles in **Figure 4**. Each row contains the worst and best fit for similar 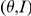-combinations. The rows follow the red circles first along the line from bottom left to top right and then the circles off the line from middle to top. Red bars denote the data generated by the spatially explicit model and the black line denotes the spatially implicit model prediction. The 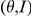-parameter values used are (from top to bottom, first across then down): 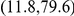, 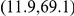, 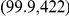, 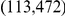, 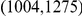, 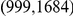, 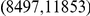, 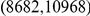, 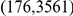, 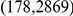, 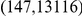, 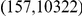.

**Figure 6:**
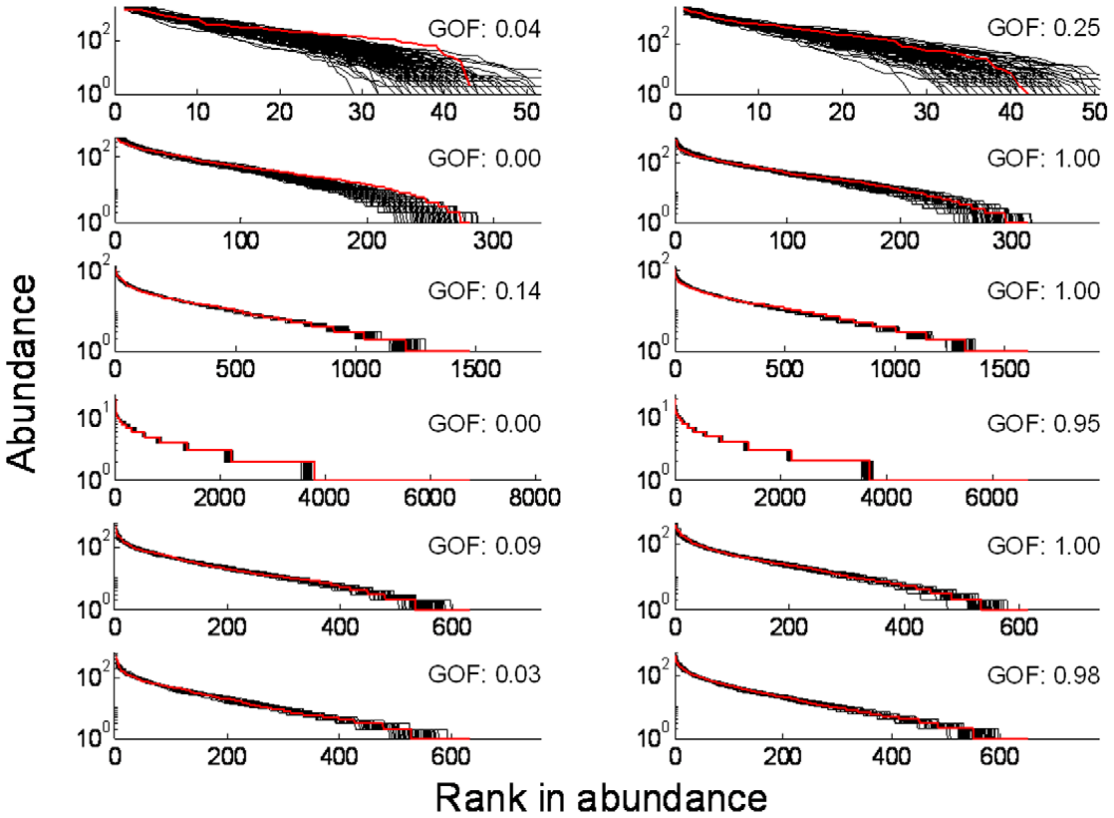
The rank abundance distributions for 100 data sets generated by the spatially implicit model (black) and for the spatially explicit data of **Figure 5** (red). The parameter values are the same as in Figure 5.

## Supporting Information

Figure S1 (0.26 MB TIF)

Figure S2 (0.35 MB TIF)

Figure S3 (0.76 MB TIF)

Figure S4 (0.63 MB TIF)
