# Supplementary figures and images for: The Spatial Limitations of Current Neutral Models of Biodiversity
Source: PLoS One. 2011 Mar 14;6(3):e14717. doi: 10.1371/journal.pone.0014717 (PMC3056651; doi:10.1371/journal.pone.0014717)

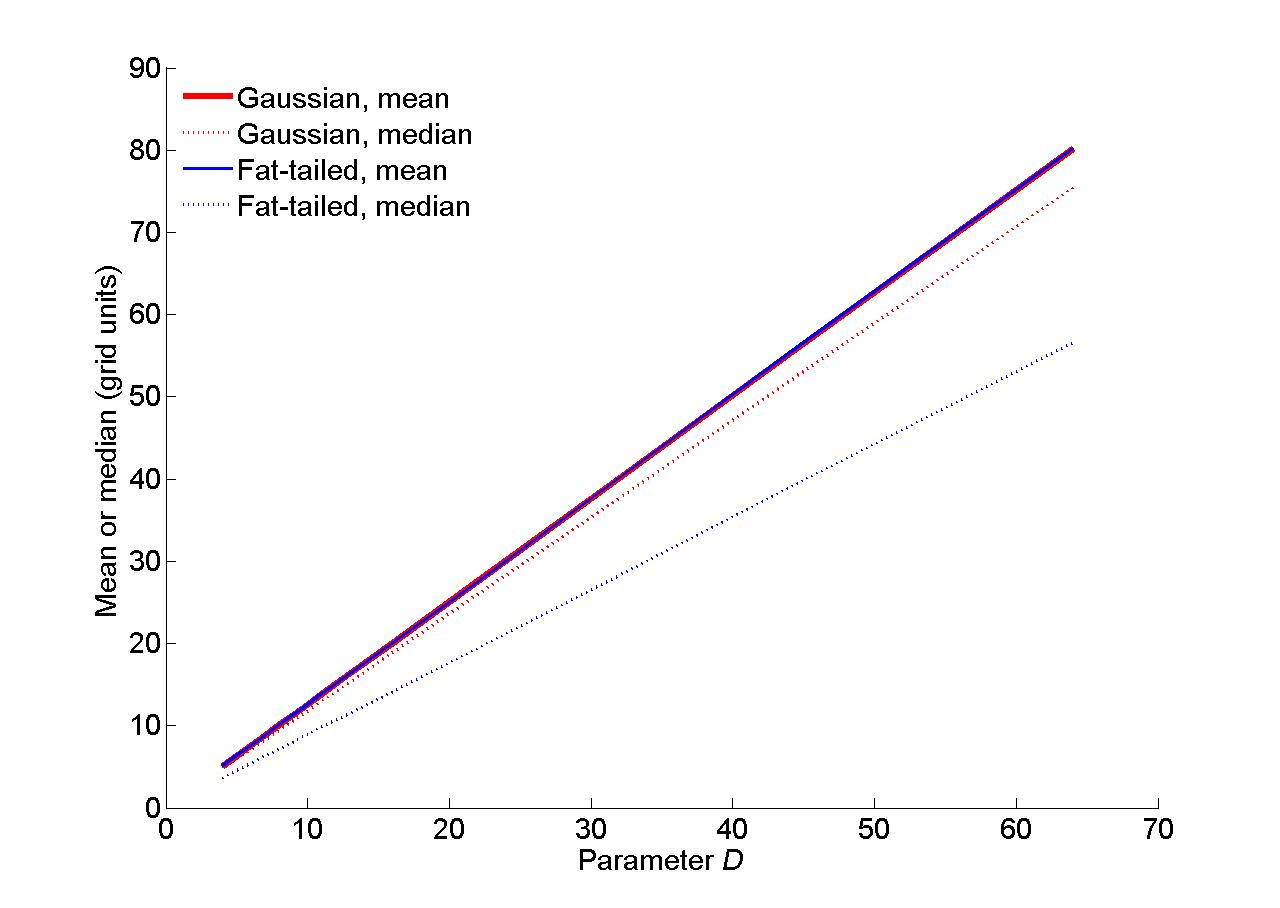

Supplement: Figure S1 — (0.26 MB TIF) [file pone.0014717.s001.tif]

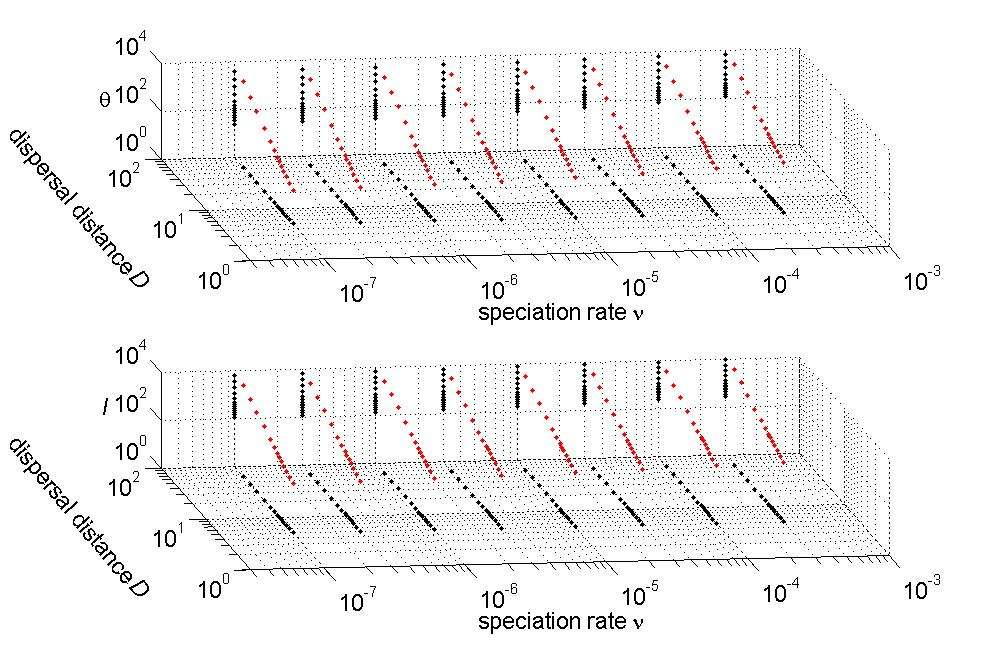

Supplement: Figure S2 — (0.35 MB TIF) [file pone.0014717.s002.tif]

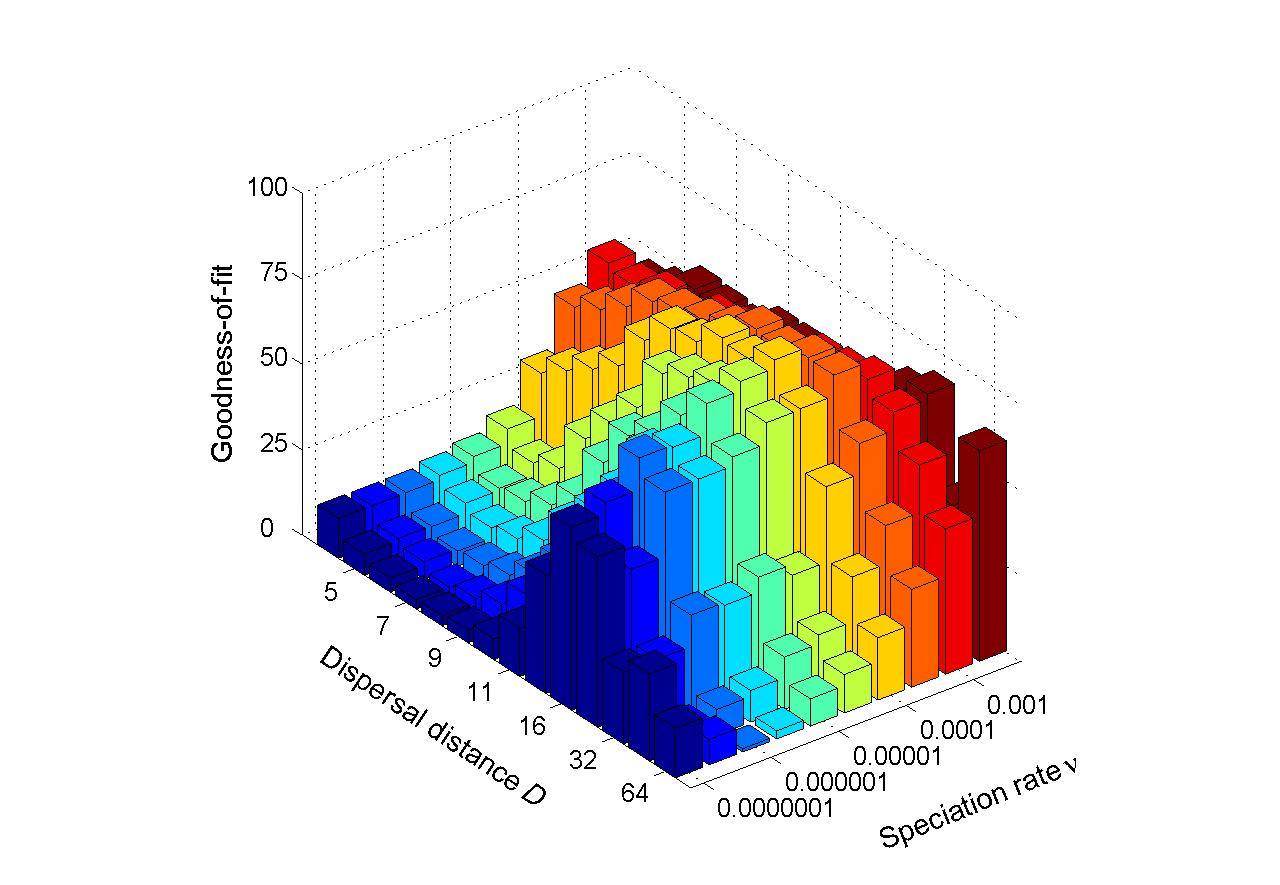

Supplement: Figure S3 — (0.76 MB TIF) [file pone.0014717.s003.tif]

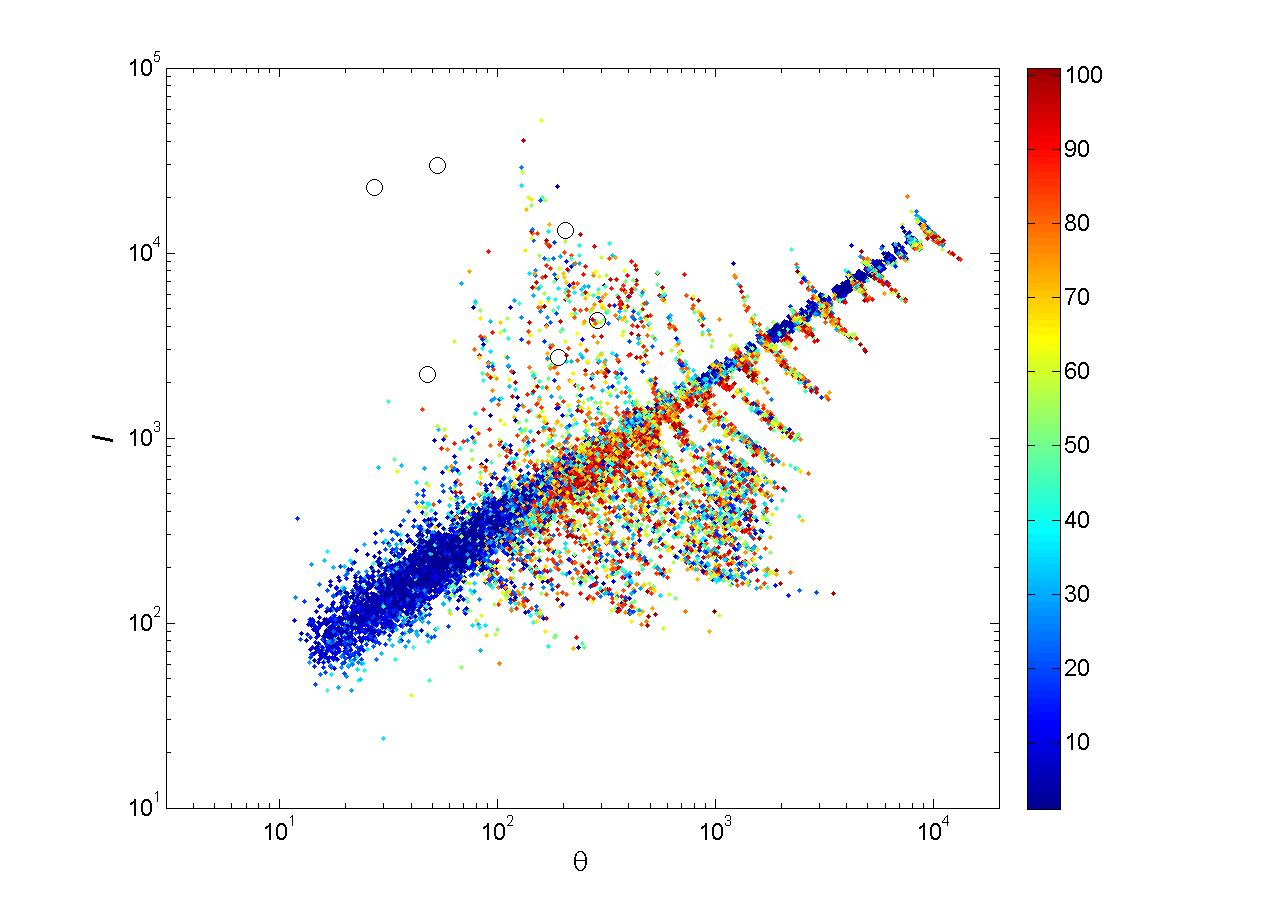

Supplement: Figure S4 — (0.63 MB TIF) [file pone.0014717.s004.tif]
